# Supplementary material for: Health Impacts from Ambient Particle Exposure in Southern Sweden
Source: Int J Environ Res Public Health. 2020 Jul 14;17(14):5064. doi: 10.3390/ijerph17145064 (PMC7400131; doi:10.3390/ijerph17145064)
Supplement: Supplementary file 1 [file ijerph-17-05064-s001.pdf]

Supplementary

Health Impacts from Ambient Particle Exposure in Southern Sweden

Ralf Rittner, Erin Flanagan, Anna Oudin and Ebba Malmqvist

Table S1. Estimated $PM_{2.5}$ exposure for the Scania population using 2011 concentrations by source.

Source	$PM_{2.5}$ (μg/m³)
Background	11.00
All local	0.88
Traffic	0.26
Small-scale heating	0.19
Shipping	0.02
Industry and energy production	0.02
Unspecified local	0.39

Table S2. Estimated $PM_{2.5}$ (μg/m³) exposure for the Scania population in 2011 by source and population subgroup.

	All local [1]	Industry and Energy Production	Shipping	Small-Scale Heating	Traffic
	Mean (min max)	Mean (min max)	Mean (min max)	Mean (min max)	Mean (min max)
Total population (N = 1,247,993)	0.88 (0.06–9.90)	0.02 (0.00–5.84)	0.02 (0.00–0.47)	0.19 (0.01–11.00)	0.26 (0.00–1.57)
**Sex**					
Men (N = 617,568)	0.87 (0.06–9.90)	0.02 (0.00–5.84)	0.02 (0.00–0.47)	0.19 (0.01–10.99)	0.26 (0.00–1.57)
Women (N = 630,425)	0.88 (0.06–9.90)	0.02 (0.00–5.84)	0.02 (0.00–0.47)	0.18 (0.01–10.99)	0.27 (0.00–1.57)
**Age Groups**					
<18 (N = 253,325)	0.94 (0.06–9.90)	0.02 (0.00–4.68)	0.02 (0.00–0.45)	0.19 (0.01–10.86)	0.24 (0.01–1.57)
18–29 (N = 202,694)	0.99 (0.06–8.89)	0.02 (0.00–5.18)	0.03 (0.00–0.47)	0.18 (0.01–10.86)	0.33 (0.01–1.57)
30–65 (N = 574,118)	0.87 (0.06–9.90)	0.02 (0.00–5.84)	0.02 (0.00–0.47)	0.18 (0.01–10.99)	0.26 (0.00–1.57)
>65 (N = 217,856)	0.83 (0.06–8.64)	0.02 (0.00–5.83)	0.03 (0.00–0.47)	0.19 (0.01–8.07)	0.24 (0.00–1.52)

[1] Total local concentration includes: industry and energy production, shipping, small-scale heating, traffic and other sources.

Table S3. Regional exposure differences of sources for a municipality with frequent small-scale heating, a harbor city and a large city, local contribution and background for 2011.

	Osby (Heating Municipality) N = 12,664	Trelleborg (Harbor City) N = 42,460	Malmö (Large City) N = 300,691
	$PM_{2.5}$ (μg/m³)		
Total local concentration [1]	1.07	0.68	1.41
Industry and energy production	0.00	0.01	0.01
Shipping	0.00	0.08	0.02
Small-scale heating	0.95	0.15	0.13
Traffic	0.05	0.12	0.58
Background	9.50	9.50	15.90

[1] Total local concentration includes: industry and energy production, shipping, small-scale heating, traffic and other sources.
